# Prediction of Major Adverse Cardiovascular Events in Patients with Peripheral Artery Disease Using Circulating Immunomodulatory Proteins

**DOI:** 10.3390/biomedicines12122842

**Published:** 2024-12-13

**Authors:** Ben Li, Farah Shaikh, Houssam Younes, Batool Abuhalimeh, Jason Chin, Khurram Rasheed, Abdelrahman Zamzam, Rawand Abdin, Mohammad Qadura

**Affiliations:** 1 Department of Surgery, University of Toronto, Toronto, ON M5S 1A1, Canada; benx.li@mail.utoronto.ca; 2 Division of Vascular Surgery, St. Michael’s Hospital, Unity Health Toronto, University of Toronto, Toronto, ON M5B 1W8, Canada; farah.shaikh@unityhealth.to (F.S.); abdelrahman.zamzam@unityhealth.to (A.Z.); 3 Institute of Medical Science, University of Toronto, Toronto, ON M5S 1A1, Canada; 4 Temerty Centre for Artificial Intelligence Research and Education in Medicine (T-CAIREM), University of Toronto, Toronto, ON M5S 1A1, Canada; 5 Heart, Vascular, & Thoracic Institute, Cleveland Clinic Abu Dhabi, Abu Dhabi 112412, United Arab Emirates; younesh@clevelandclinicabudhabi.ae (H.Y.); abuhalb@clevelandclinicabudhabi.ae (B.A.); chinj@clevelandclinicabudhabi.ae (J.C.); rasheedk@clevelandclinicabudhabi.ae (K.R.); 6 Department of Medicine, McMaster University, Hamilton, ON L8S 4L8, Canada; rawand.abdin@medportal.ca; 7 Li Ka Shing Knowledge Institute, St. Michael’s Hospital, Unity Health Toronto, University of Toronto, Toronto, ON M5B 1W8, Canada

**Keywords:** immunomodulatory proteins, galectin-1, galectin-9, alpha-1-microglobulin, major adverse cardiovascular events, prognosis, peripheral artery disease

## Abstract

**Background/Objectives:** The leading cause of death for people with peripheral artery disease (PAD) is major adverse cardiovascular events (MACE), including heart attacks and strokes. However, research into biomarkers that could help predict MACE in patients with PAD has been limited. Immunomodulatory proteins are known to significantly influence systemic atherosclerosis, suggesting they could be useful prognostic indicators for MACE in patients with PAD. In this study, we evaluated a broad panel of immunomodulatory proteins to identify those linked to MACE in individuals with PAD. **Methods:** We conducted a prognostic study involving a prospectively recruited cohort of 406 patients consisting of 254 with PAD and 152 without PAD. At the baseline, we measured the plasma concentrations of 17 circulating immunomodulatory proteins and followed the cohort for two years. The primary outcome was 2-year MACE, a composite of myocardial infarction, stroke, or death. Plasma protein concentrations were compared between patients with PAD with and without 2-year MACE using Mann–Whitney U tests. We further examined the prognostic potential of differentially expressed proteins through a Cox proportional hazards analysis, determining their independent associations with 2-year MACE while controlling for all the baseline demographic and clinical characteristics, including the existing coronary artery and cerebrovascular diseases. Additionally, A Kaplan–Meier analysis was performed to evaluate the 2-year freedom from MACE in patients with low versus high levels of the differentially expressed proteins based on the median plasma concentrations. **Results:** The mean age of the cohort was 68.8 years (SD 11.1), with 134 patients (33%) being female. During the two-year follow-up, 63 individuals (16%) developed MACE. The following proteins were significantly elevated in patients with PAD who experienced 2-year MACE compared to those who did not: galectin-1 (0.17 [SD 0.06] vs. 0.10 [SD 0.07] pg/mL, *p* = 0.012), alpha-1-microglobulin (16.68 [SD 7.48] vs. 14.74 [SD 6.71] pg/mL, *p* = 0.019), and galectin-9 (0.14 [SD 0.09] vs. 0.09 [SD 0.05] pg/mL, *p* = 0.033). The Cox proportional hazards analysis indicated that these three proteins were independently associated with 2-year MACE after adjusting for all the baseline demographic and clinical factors: galectin-1 (HR 1.45 [95% CI 1.09–1.92], *p* = 0.019), alpha-1-microglobulin (HR 1.31 [95% CI 1.06–1.63], *p* = 0.013), and galectin-9 (HR 1.35 [95% CI 1.02–1.78], *p* = 0.028). Over the two-year follow-up, patients with higher levels of galectin-1, galectin-9, and alpha-1-microglobulin had a lower freedom from MACE. Additional analysis showed that these three proteins were not significantly associated with 2-year MACE in patients without PAD. **Conclusions:** Among the 17 immunomodulatory proteins evaluated, galectin-1, galectin-9, and alpha-1-microglobulin were found to be independently and specifically associated with 2-year MACE in patients with PAD. Assessing the plasma concentrations of these proteins can aid in risk stratification for MACE in patients with PAD, helping to inform clinical decisions regarding multidisciplinary referrals to cardiologists, neurologists, and vascular medicine specialists. This information can also guide the aggressiveness of medical management, ultimately improving cardiovascular outcomes for patients with PAD.

## 1. Introduction

Peripheral artery disease (PAD) involves atherosclerosis in the lower extremity arteries and affects over 200 million people worldwide [1]. While there is a notable connection between PAD and major adverse limb events leading to amputation, the majority of patients with PAD ultimately die from major adverse cardiovascular events (MACE), such as myocardial infarction (MI) and stroke [2]. This is largely due to the significant link between PAD and both coronary artery disease (CAD) and cerebrovascular disease (CVD) [3]. The underlying mechanism for this relationship involves systemic atherosclerosis driven by common risk factors such as advanced age, hypertension, diabetes, dyslipidemia, and smoking [4]. Therefore, identifying patients with PAD at high risk for MACE is crucial for ensuring they receive multidisciplinary evaluations and appropriate cardiovascular risk-reduction treatments [5]. One approach to address this issue is to discover novel biomarkers that can predict MACE in patients with PAD. Our group has previously identified several biomarkers capable of predicting major adverse limb events (MALEs) in patients with PAD, including fatty acid binding proteins [6,7,8,9,10], inflammatory proteins [11], and Cystatin C [12]. However, there has been limited investigation into biomarkers that can predict MACE in patients with PAD.

The immune system has been demonstrated to play an important role in cardiovascular diseases due to its mechanistic roles in systemic inflammation, atherosclerosis, and thrombosis. Therefore, immunomodulatory proteins may act as biomarkers for MACE in patients with PAD given their potential involvement in various pathways contributing to systemic atherosclerosis [13]. Immunomodulatory proteins have been demonstrated to play a role in the development and progression of cardiovascular diseases, including galectin-1, galectin-9, and alpha-1-microglobulin [14,15,16]. In fact, over 10 immunomodulatory proteins have been demonstrated to be associated with PAD, CAD, and CVD [17,18,19,20,21,22]. The selection of these specific 17 immunomodulatory proteins for analysis in this study stems from their extensive investigation and robust associations with cardiovascular diseases, indicating their potential relevance in predicting MACE in patients with PAD [17,18,19,20,21,22]. While previous studies have shown correlations between these proteins and cardiovascular conditions, few have specifically explored their prognostic implications for MACE in patients with PAD [17,18,19,20,21,22]. The aim of this study was to identify prognostic biomarkers that can predict MACE in patients with PAD from a large panel of immunomodulatory proteins, ultimately seeking to identify high-risk individuals who may benefit from more aggressive medical management for systemic atherosclerosis to prevent adverse events.

## 2. Materials and Methods

### 2.1. Ethics

The research ethics board at Unity Health Toronto, University of Toronto, ON, Canada, granted approval for the study on 8 February 2017 (REB # 16-365). Prior to participating, all individuals provided informed consent, and all the procedures were carried out in full compliance with the principles set forth in the Declaration of Helsinki [23].

### 2.2. Study Design

This study was a prognostic analysis, and the findings were reported in accordance with the Transparent Reporting of a Multivariable Prediction Model for Individual Prognosis or Diagnosis + Artificial Intelligence (TRIPOD+AI) statement [24].

### 2.3. Cohort Recruitment

This study involved the prospective recruitment of consecutive patients, both with and without PAD, who received care at St. Michael’s Hospital ambulatory vascular clinics from January 2018–August 2019. PAD was diagnosed using an Ankle–Brachial Index (ABI) of less than 0.9 or a Toe–Brachial Index (TBI) of less than 0.7, along with absent or diminished pedal pulses [25]. Conversely, non-PAD was characterized by an ABI of 0.9 or higher, a TBI of 0.7 or higher, and normal pedal pulses [25]. Patients with acute limb ischemia, acute coronary syndrome, or elevated troponin levels within the past three months were excluded from the study.

### 2.4. Baseline Demographic and Clinical Characteristics

The baseline characteristics recorded in this study included age, sex, hypertension (defined as a systolic blood pressure of ≥130 mmHg, diastolic blood pressure of ≥80 mmHg, or currently receiving blood pressure-lowering medication [26,27]), dyslipidemia (total cholesterol > 5.2 mmol/L, triglycerides > 1.7 mmol/L, or currently on lipid-lowering therapy [26,27]), diabetes (hemoglobin A1c ≥ 6.5% or currently taking antidiabetic medication [26,27]), smoking history (both current and past), and the presence of CAD, congestive heart failure (CHF), and a history of stroke. Definitions for the cardiovascular risk factors were derived from the guidelines from the American College of Cardiology [26,27].

### 2.5. Quantification of Plasma Protein Concentrations

Blood samples were collected from the patients, and the plasma concentrations of 17 immunomodulatory proteins were measured in duplicate using a commercially available LUMINEX assay (Bio-Techne, Minneapolis, MN, USA) following the manufacturer’s guidelines [28]. The following proteins were chosen based on their involvement in various immunomodulatory processes associated with systemic atherosclerosis and important associations with cardiovascular diseases: galectin-1, alpha-1-microglobulin, galectin-9, chemerin, interleukin-2 (IL-2), cluster of differentiation 40 (CD40), A proliferation-inducing ligand (APRIL)/tumor necrosis factor ligand superfamily member 13 (TNFSF13), activated leukocyte cell adhesion molecule (ALCAM)/CD166, cathepsin-S, CD40 ligand, tumor necrosis factor receptor II (TNFRII), aggrecan, Epithelial Cellular Adhesion Molecule (EpCAM)/troponin 1 (TROP1), Receptor for Advanced Glycation Endproducts (RAGE), CXC chemokine ligand 6 (CXCL6), TNFRSF9/CD137, and IL-33. The analysis of multiple immunomodulatory proteins aims to identify novel biomarkers for PAD. Before sample analysis, Fluidics Verification and Calibration bead kits (Luminex Corp) [29] were used to calibrate the MagPix analyzer (Luminex Corp; Austin, TX, USA) [30]. To reduce inter-assay variability, all the sample analyses were performed on the same day. The intra-assay and inter-assay coefficients of variability for the samples were both under 10%. At least 50 beads for each protein were collected and analyzed using the Luminex xPonent software version 4.3 [31].

### 2.6. Follow-Up and Outcomes

Routine outpatient clinic visits took place at 1 and 2 years following the baseline assessment, with additional appointments as needed based on clinical complications. The primary outcome of interest was the occurrence of MACE over 2 years, which included a composite of stroke, myocardial infarction, and death, captured through direct clinical follow-up. Myocardial infarction was defined as a rise and/or fall in cardiac troponin values with at least 1 value above the 99th percentile upper reference limit and at least 1 of the following: (a) symptoms of myocardial ischemia, (b) new ischemic electrocardiogram changes, (c) the development of pathological Q waves, (d) imaging evidence of the new loss of viable myocardium or new regional wall motion abnormality in a pattern consistent with an ischemic etiology, or (e) the identification of a coronary thrombus by angiography or autopsy [32]. This definition is based on the guidelines by the European Society of Cardiology, American College of Cardiology, American Heart Association, and World Heart Federation [32]. Stroke was defined as brain, spinal cord, or retinal cell death attributable to ischemia based on (a) pathological, imaging, or other objective evidence of cerebral, spinal cord, or retinal focal ischemic injury in a defined vascular distribution, or (b) the clinical evidence of cerebral, spinal cord, or retinal focal ischemic injury based on symptoms persisting ≥ 24 h or until death, and other etiologies excluded [33]. This definition is based on the guidelines by the American Heart Association and American Stroke Association [33]. Death was defined as all-cause mortality.

### 2.7. Statistical Analysis

The demographic and clinical characteristics of our cohort were summarized using means and standard deviations (SDs) or counts and proportions. Baseline differences between groups were evaluated with independent t-tests for continuous variables and chi-square tests for categorical variables. Plasma protein concentrations were analyzed between patients with PAD with and without 2-year MACE using Mann–Whitney U tests. Proteins that showed differential expression in patients with PAD with 2-year MACE compared to those without 2-year MACE were further examined for their prognostic potential. Rather than performing a propensity score-matched analysis, which can reduce sample size important for the assessment of prognostic potential, we performed a multivariable Cox proportional hazards analysis, which controls for baseline factors while maintaining the sample size. Specifically, associations between these proteins and 2-year MACE were assessed using the Cox proportional hazards analysis, adjusting for age, sex, hypertension, dyslipidemia, diabetes, past/current smoking, CHF, CAD, history of stroke, and PAD status, accounting for the competing risk of death using the subdistribution hazard function defined by Fine and Gray [34]. These variables were selected for the multivariable Cox proportional hazards model based on testing for co-linearity (variance inflation factor < 5) and the criteria for the proportionality of hazards, whereby the ratio of hazard rates for the two groups was constant over time, and it was fulfilled as determined by the weighted residual score test defined by Grambsch and Therneau [35]. The cohort was then stratified into low vs. high levels of differentially expressed proteins based on the median plasma concentrations (0.16 pg/mL for galectin-1, 15.1 pg/mL for alpha-1-microglobulin, and 0.10 pg/mL for galectin-9). Freedom from MACE over 2 years was analyzed using Kaplan–Meier curves, with comparisons made through the Cox proportional hazards analysis, adjusting for all the baseline characteristics, a validated approach for assessing the clinical importance of prognostic biomarkers [36,37]. This stratified analysis aimed to determine the clinical prognostic value of each significant protein, helping clinicians understand how a patient’s risk of MACE differs based on low vs. high circulating protein levels. The following statistical tests were performed to assess the prognostic value of the identified biomarkers: receiver operating characteristic (ROC) curve analysis, C-statistic, integrated discrimination improvement (IDI), and net reclassification improvement (NRI) [38,39,40]. The C-statistic for predicting 2-year MACE was calculated for the baseline clinical characteristics alone (age, sex, hypertension, dyslipidemia, diabetes, past/current smoking, CHF, CAD, history of stroke, and PAD status) and for the baseline clinical characteristics plus each of the 3 identified biomarkers (galectin-1, alpha-1-microglobulin, and galectin-9). The reason for evaluating the proteins in this fashion is to understand the relative importance of each biomarker in contributing to 2-year MACE predictions compared to the baseline clinical characteristics alone. C-statistics were compared using DeLong’s test [41]. Additionally, NRI and IDI are different measurements that quantify how much the addition of each biomarker to the baseline clinical features improves model performance for predicting 2-year MACE (e.g., improvement in model discrimination between presence vs. absence of 2-year MACE) [40]. Statistical significance was set at a two-tailed *p* < 0.05. All the continuous variables examined had normal distributions, and statistical significance was verified, even for the variables with overlapping standard deviations, given the sufficient sample size and the difference between means. Indeed, Krzywinski and Altman showed that statistical significance can be achieved even with overlapping standard deviations [42]. All the analyses were conducted using the SPSS software version 23 (SPSS Inc., Chicago, IL, USA) [43].

## 3. Results

### 3.1. Patients

A total of 406 patients participated in the study, including 254 with PAD and 152 without PAD. The patients with PAD were older, with a mean age of 71 (SD 10) compared to 65 (SD 13) years in the non-PAD group (*p* < 0.001). They also had higher rates of cardiovascular comorbidities (Table 1). At discharge, most patients were on risk-reduction medications including acetylsalicylic acid (92%) and statins (90%), with no differences between the groups.

### 3.2. Plasma Concentrations of Immunomodulatory Proteins

Among the 17 immunomodulatory proteins analyzed, three were significantly elevated in the patients with PAD who experienced 2-year MACE compared to those who did not: galectin-1, alpha-1-microglobulin, and galectin-9 (Table 2). Given the elevated plasma concentrations of galectin-1, alpha-1-microglobulin, and galectin-9 in the patients with PAD who developed 2-year MACE, their prognostic potential was further explored in this study.

### 3.3. Major Adverse Cardiovascular Events

Throughout the 2-year follow-up period, MACE occurred in 63 individuals (16%), with the following breakdown: MI occurred in 51 patients (13%), stroke in 17 patients (4%), and death in 5 patients (1%). There were no significant differences in the 2-year event rates between the patients with PAD and those without PAD (Table 3).

### 3.4. Associations Between Immunomodulatory Proteins and Major Adverse Cardiovascular Events in Patients with Peripheral Artery Disease

Plasma concentrations of all three immunomodulatory proteins (galectin-1, alpha-1-microglobulin, and galectin-9) were independently associated with 2-year MACE in the patients with PAD even after adjusting for all the baseline demographic and clinical characteristics (Table 4). Additional analysis indicated no significant associations between these proteins and 2-year MACE in the patients without PAD. This demonstrates that galectin-1, alpha-1-microglobulin, and galectin-9 specifically predict 2-year MACE in patients with PAD. Interaction testing demonstrated a significant association between the elevated levels of these 3 proteins and the risk of 2-year MACE in the patients with PAD (*p* < 0.05).

### 3.5. Kaplan–Meier Analysis

**Figure 1:**
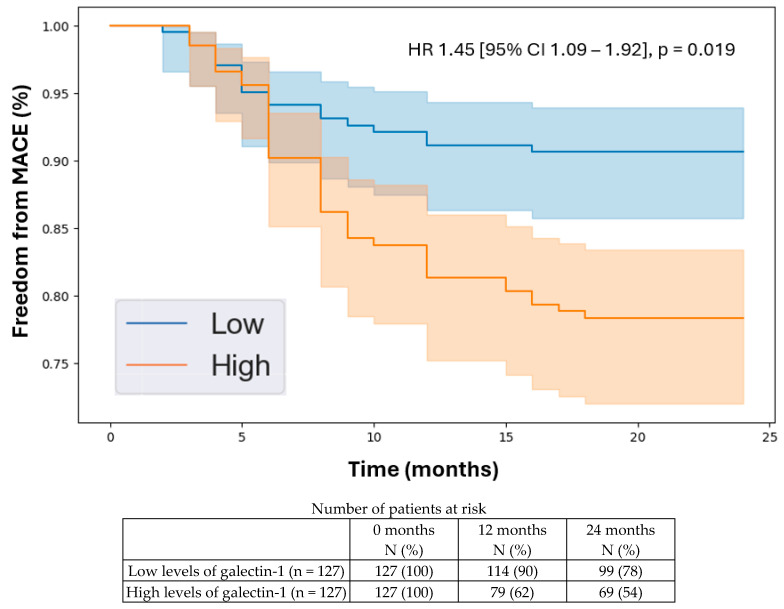
Kaplan–Meier analysis of freedom from major adverse cardiovascular events in patients with peripheral artery disease stratified by low vs. high levels of galectin-1 based on the median plasma concentration in the cohort (0.16 pg/mL). Cox proportional hazards analysis adjusted for age, sex, hypertension, dyslipidemia, diabetes, past/current smoking, congestive heart failure, coronary artery disease, and previous stroke. Abbreviations: MACE (major adverse cardiovascular event); HR (hazard ratio); CI (confidence interval).

**Figure 2:**
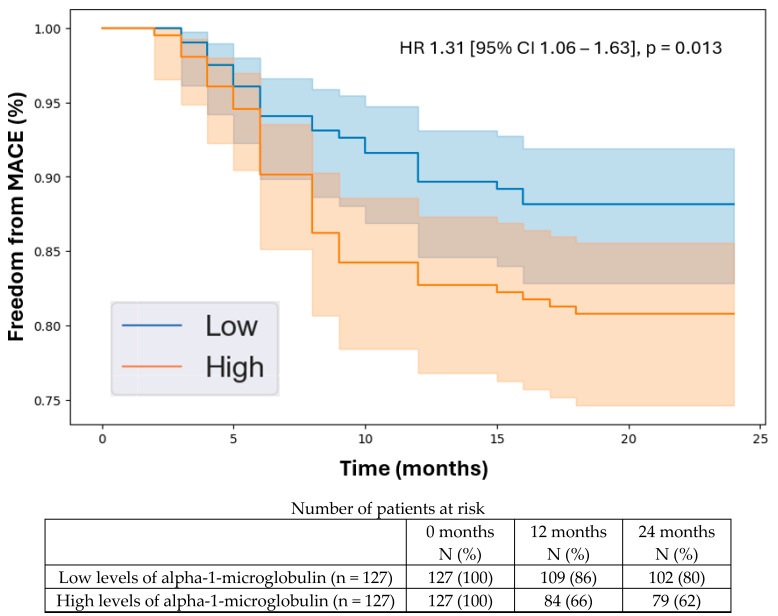
Kaplan–Meier analysis of freedom from major adverse cardiovascular events in patients with peripheral artery disease stratified by low vs. high levels of alpha-1-microglobulin based on the median plasma concentration in the cohort (15.1 pg/mL). Cox proportional hazards analysis adjusted for age, sex, hypertension, dyslipidemia, diabetes, past/current smoking, congestive heart failure, coronary artery disease, and previous stroke. Abbreviations: MACE (major adverse cardiovascular event); HR (hazard ratio); CI (confidence interval).

**Figure 3:**
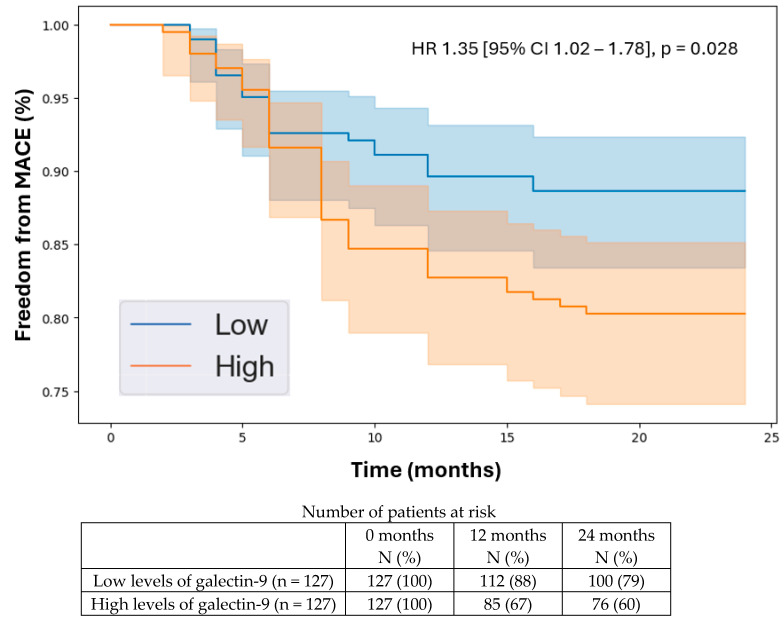
Kaplan–Meier analysis of freedom from major adverse cardiovascular events in patients with peripheral artery disease stratified by low vs. high levels of galectin-9 based on the median plasma concentration in the cohort (0.10 pg/mL). Cox proportional hazards analysis adjusted for age, sex, hypertension, dyslipidemia, diabetes, past/current smoking, congestive heart failure, coronary artery disease, and previous stroke. Abbreviations: MACE (major adverse cardiovascular event); HR (hazard ratio); CI (confidence interval).

The Kaplan–Meier analysis indicated that the patients with higher levels of the identified proteins experienced a lower freedom from MACE over the 2-year follow-up period. Specifically, the hazard ratios were as follows: galectin-1 (HR 1.45 [95% CI 1.09–1.92], *p* = 0.019, Figure 1), alpha-1-microglobulin (HR 1.31 [95% CI 1.06–1.63], *p* = 0.013, Figure 2), and galectin-9 (HR 1.35 [95% CI 1.02–1.78], *p* = 0.028, Figure 3). The C-statistic of the model with the baseline variables alone for predicting 2-year MACE was 0.70 (95% CI 0.67–0.72). The C-statistics of the models with the baseline variables plus individual biomarkers were the following: galectin-1, 0.88 (95% CI 0.86–0.90, NRI 0.43, IDI 0.06, *p* < 0.001); alpha-1-microglobulin, 0.86 (95% CI 0.83–0.88, NRI 0.42, IDI 0.06, *p* < 0.001); and galectin-9, 0.82 (95% CI 0.79–0.85, NRI 0.40, IDI 0.05, *p* = 0.002). These results demonstrate that the addition of each of the biomarkers significantly improved model performance for predicting 2-year MACE compared to the baseline clinical characteristics alone. A subgroup analysis demonstrated similar prognostic values of these three biomarkers in the patients with PAD and CAD and those with PAD without existing CAD at the baseline, demonstrating the specificity of these proteins for predicting 2-year MACE in patients with PAD with or without existing CAD.

## 4. Discussion

### 4.1. Summary of Findings

In this study, we identified galectin-1, alpha-1-microglobulin, and galectin-9 as the immunomodulatory proteins that are independently associated with 2-year MACE in patients with PAD, thereby acting as potential prognostic biomarkers. Several key findings emerged from our analysis. First, from the 17 circulating immunomodulatory proteins analyzed, we found that galectin-1, alpha-1-microglobulin, and galectin-9 were the only proteins that were significantly elevated in the patients with PAD with 2-year MACE compared to the patients with PAD without 2-year MACE. Second, we demonstrated independent associations between galectin-1, alpha-1-microglobulin, and galectin-9 and 2-year MACE in the patients with PAD after controlling for the baseline demographic and clinical characteristics, including existing CAD and CVD. Importantly, we showed that there were no significant associations between these three proteins and 2-year MACE in the patients without PAD, suggesting that these biomarkers specifically predict MACE in patients with PAD. Third, we used the median plasma concentrations of galectin-1, alpha-1-microglobulin, and galectin-9 to stratify the patients into low vs. high levels of each protein. Using the Kaplan–Meier analysis, we demonstrated that the patients with high levels of each protein were more likely to develop MACE over a 2-year period. This demonstrates the clinical relevance of galectin-1, alpha-1-microglobulin, and galectin-9 in helping clinicians understand the future trajectory of their patients with PAD in terms of MACE risk. This can help them identify high-risk patients and ensure that they receive appropriate cardiovascular risk-reduction treatment strategies. Given the significance of galectin-1, alpha-1-microglobulin, and galectin-9 in predicting MACE specifically for patients with PAD, further basic science and translational research is warranted to elucidate the biological relationships between these proteins and cardiovascular disease development/progression in patients with PAD with the goal of informing targeted therapeutics.

### 4.2. Comparison to Existing Literature

Seropian et al. (2018) reviewed the literature on galectin-1 as an emerging mediator of cardiovascular inflammation and demonstrated the important role of this protein in cardiovascular diseases including acute MI, heart failure, and ischemic stroke through its involvement in immune cell homeostasis and chronic inflammation [14]. Specifically, Chou and colleagues (2020) showed that galectin-1 is associated with the severity of CAD and adverse cardiovascular events in patients undergoing coronary angiography [44]. Similarly, Krautter and colleagues (2022) demonstrated the pathological role of galectin-9 in promoting monocyte recruitment and atherosclerotic plaque progression in patients with PAD [45]. Elsewhere, Cui et al. (2021) showed that urinary alpha-1-microglobulin is a predictor for in-hospital mortality in patients with ST-segment elevation MI [16]. Our findings corroborate the literature regarding the important roles of galectin-1, galectin-9, and alpha-1-microglobulin in cardiovascular diseases. We additionally demonstrate the prognostic value of these proteins in predicting MACE specifically in patients with PAD, which has not been previously investigated. Li et al. (2022) previously demonstrated that inflammatory proteins are associated with major adverse limb events in patients with PAD [11]. In this study, by further demonstrating that immunomodulatory proteins can predict MACE in patients with PAD, we additionally show the importance of these proteins in the biological pathways related to the progression of systemic cardiovascular diseases. Therefore, our work highlights the importance of further investigating the mechanistic relationship between immunomodulatory proteins and the combination of PAD, CAD, and CVD with the goal of strengthening our understanding of these pathologies and potentially unveiling novel therapeutic strategies.

### 4.3. Explanation of Findings

There are several potential explanations for our findings. First, galectin-1 belongs to the family of galectins, which are carbohydrate-binding proteins with an affinity for beta-galactosides [46]. Galectin-1 is differentially expressed by various normal and pathological tissues and appears to be functionally polyvalent with a wide range of biological activity [46]. Accumulating evidence demonstrates that galectin-1 and its ligands are important regulators of immune responses such as T-cell homeostasis and survival, T-cell immune disorders, and chronic inflammation [46]. Given that systemic atherosclerosis is primarily a disease of chronic inflammation, these mechanisms may explain the important role of galectin-1 in PAD, CAD, and CVD [47]. Specifically, Chou and colleagues (2020) showed that individuals with higher galectin-1 levels had a greater prevalence of PAD, CVD, and CAD [44]. Similarly, Ou et al. (2022) demonstrated that galectin-1 alleviates myocardial ischemia–reperfusion injury by reducing the inflammation and apoptosis of cardiomyocytes [48]. These studies may explain the mechanism through which galectin-1 predicted MACE in patients with PAD in our cohort. Second, galectin-9 belongs to the same protein family as galectin-1; however, it is a unique member in that it has two non-homologous carbohydrate recognition domains joined by a linker peptide sequence of variable lengths, thereby generating isoforms with distinct properties and functions [49]. Specifically, it plays an important role in both the physiological and pathological settings related to development and immunity [49]. Mansour and colleagues (2022) showed that galectin-9 supports primary T cell trans-endothelial migration in a glycan- and integrin-dependent manner [50]. This is particularly relevant in the setting of chronic inflammation leading to atherosclerosis and vascular endothelial dysfunction in cardiovascular disorders [50]. Other groups have demonstrated the important interplay of galectins-1 and 9 in the immune-inflammatory response underlying cardiovascular and metabolic disease [51]. These studies may explain why both galectins-1 and -9 were important predictors of MACE in patients with PAD. Third, alpha-1-microglobulin is a small protein (26 kDa) and one of the three original members of the lipocalin superfamily, discovered in human urine over 40 years ago [52]. It is distributed in the plasma and extravascular compartments of all organs [52]. Alpha-1-microglobulin has a free cysteine side-chain located in a flexible loop, giving it protein reductase and dehydrogenase properties with a broad biological substrate specificity [52]. Amatruda and colleagues (2021) demonstrated that urine alpha-1-microglobulin levels are associated with acute kidney injury, mortality, and other adverse events following cardiac surgery [53]. Similarly, Ishiwata et al. (2021) showed that in patients hospitalized due to acute heart failure, urinary alpha-1-microglobulin was associated with kidney dysfunction and all-cause mortality [54]. These studies highlight the potential mechanisms through which alpha-1-microglobulin was associated with MACE in our PAD cohort. Taken together, these findings explain the biological mechanisms through which galectin-1, galectin-9, and alpha-1-microglobulin are related to cardiovascular disease progression, and therefore, may act as prognostic biomarkers for MACE in patients with PAD.

### 4.4. Implications

Our study provides practical implications for clinical decision making in patients with PAD. The measurement of galectin-1, galectin-9, and alpha-1-microglobulin can effectively determine the risk of MACE in patients with PAD, which is especially valuable in family practice settings. General practitioners can incorporate these findings by measuring the plasma levels of these proteins to assess MACE risk in patients with PAD [55]. Individuals screening positive for being at high risk of MACE can then be referred for further multidisciplinary evaluation by cardiologists, neurologists, and vascular medicine specialists [56]. In contrast, patients deemed low risk can continue care with their family physician, focusing on optimizing risk factors through management strategies such as acetylsalicylic acid (ASA), statins, and lifestyle modifications [57]. After referral, specialists can utilize our findings to tailor treatment approaches based on the predicted MACE risk over two years. For instance, adding low-dose rivaroxaban to ASA has been shown to improve cardiovascular outcomes in patients with stable PAD or CAD [58]. Moreover, the angiographic assessments of coronary and cerebrovascular arteries in high-risk patients can enable the early identification of significant atherosclerotic plaques that may require intervention [59]. Overall, our research has the potential to enhance care for patients with PAD in both generalist and specialist settings by facilitating the early identification of high-risk individuals, guiding the referral process, and supporting informed clinical decision-making regarding treatment aggressiveness. This approach can help reduce unnecessary referrals, improve cardiovascular outcomes, and lower healthcare costs [60].

### 4.5. Limitations

Our study has several limitations. Firstly, it was conducted at a single center, which necessitates further validation across multiple institutions to assess the generalizability of our findings. Secondly, the outcomes reported were based on a 2-year follow-up period; longer-term follow-up is essential to fully understand the prognostic value of the proteins, particularly given the chronic nature of PAD, CAD, and CVD. Thirdly, this study was insufficiently powered to assess the associations between the biomarkers and the individual endpoints of MACE. Future studies with larger sample sizes and number of events are needed to determine the prognostic value of these biomarkers for individual MACE components. Fourthly, MACE rather than MALE was the primary endpoint for this study as biomarkers for MALE have been previously investigated by our group [6,7,8,9,10,11,12]. Additionally, patients with PAD, rather than atherosclerotic cardiovascular disease (ASCVD), were the primary cohort for this study. Future investigations of prognostic biomarkers for MACE in patients with ASCVD would provide additional clinical utility. Fifthly, the measurement of plasma galectin-1, galectin-9, and alpha-1-microglobulin levels is primarily conducted in research settings. Additional translational research and implementation science are required to establish the clinical utility and feasibility of incorporating these protein measurements into routine care for patients with PAD.

## 5. Conclusions

In this study, we identified galectin-1, galectin-9, and alpha-1-microglobulin as the circulating immunomodulatory proteins that are independently associated with 2-year MACE in patients with PAD, thereby acting as potential biomarkers for systemic atherosclerosis. Importantly, these proteins predicted MACE risk in patients with PAD, but not in patients without PAD, highlighting the specificity of these biomarkers for the PAD population. Our work holds promise for MACE risk stratification in patients with PAD, offering improvements in targeted cardiovascular risk reduction strategies in patients with PAD. Specifically, high-risk patients can be referred for further multidisciplinary evaluation by cardiologists, neurologists, and vascular medicine specialists and may benefit from more aggressive medical interventions. This is particularly important given that most patients with PAD will die from MIs and strokes. Furthermore, our results highlight the need for basic and translational research to explore the mechanistic relationships between galectin-1, galectin-9, and alpha-1-microglobulin and the development and progression of systemic atherosclerosis. Such research may enhance our understanding of the underlying pathogenesis of PAD, CAD, and CVD, thereby informing targeted therapeutic strategies.

## Figures and Tables

**Table 1:** Baseline demographic and clinical characteristics of patients with and without peripheral artery disease.

		Non-PAD (n = 152)	PAD (n = 254)	*p*-Value
Age, years, mean (SD)		65 (13)	71 (10)	<0.001
Sex, n (%)	Male	97 (64)	175 (69)	0.344
	Female	55 (36)	79 (31)	
Hypertension, n (%)		90 (59)	215 (85)	<0.001
Dyslipidemia, n (%)		100 (66)	209 (82)	<0.001
Diabetes, n (%)		27 (18)	120 (47)	<0.001
Smoking, n (%)	Past	64 (42)	147 (58)	<0.001
	Current	28 (18)	60 (24)	
Congestive heart failure, n (%)		2 (1)	12 (5)	0.124
Coronary artery disease, n (%)		36 (24)	99 (39)	0.002
Previous stroke, n (%)		16 (11)	50 (20)	0.035

Abbreviations: PAD (peripheral artery disease); SD (standard deviation).

**Table 2:** Plasma concentrations of immunomodulatory proteins in patients with peripheral artery disease with and without major adverse cardiovascular events.

	No MACE (n = 208)	MACE (n = 46)	*p*-Value
Galectin-1	0.10 (0.07)	0.17 (0.06)	**0.012**
Alpha-1-Microglobulin	14.74 (6.71)	16.68 (7.48)	**0.019**
Galectin-9	0.09 (0.05)	0.14 (0.09)	**0.033**
Chemerin	8.87 (4.06)	10.61 (10.90)	0.061
IL-2	0.12 (0.95)	0.07 (1.03)	0.072
CD40	0.11 (0.50)	0.06 (1.20)	0.109
APRIL/TNFSF13	0.10 (0.75)	0.06 (1.12)	0.122
ALCAM/CD166	11.99 (4.75)	12.72 (5.79)	0.195
Cathepsin-S	0.08 (1.04)	0.05 (0.98)	0.196
CD40 Ligand	0.06 (0.90)	0.04 (1.06)	0.331
TNFRII	0.06 (0.91)	0.04 (1.05)	0.352
Aggrecan	2.20 (2.28)	2.42 (2.40)	0.370
EpCAM/TROP1	946.44 (857.51)	1.01 (741.51)	0.415
RAGE	2.40 (1.32)	2.51 (1.68)	0.491
CXCL6	250.57 (220.84)	237.62 (165.17)	0.500
TNFRSF9/CD137	78.02 (87.79)	80.26 (68.53)	0.780
IL-33	26.67 (32.76)	26.92 (15.68)	0.926

Protein concentrations reported in pg/mL. Bolded *p*-value represents statistical significance (*p* < 0.05). Abbreviations: interleukin (IL); cluster of differentiation (CD); a proliferation-inducing ligand (APRIL); tumor necrosis factor ligand superfamily member (TNFSF); activated leukocyte cell adhesion molecule (ALCAM); tumor necrosis factor receptor II (TNFRII); Epithelial Cellular Adhesion Molecule (EpCAM); troponin 1 (TROP1); Receptor for Advanced Glycation Endproducts (RAGE); CXC chemokine ligand 6 (CXCL6).

**Table 3:** Major adverse cardiovascular events over 2 years in patients with and without peripheral artery disease.

Event, n (%)	Non-PAD (n = 152)	PAD (n = 254)	*p*-Value
Major adverse cardiovascular event	17 (11)	46 (18)	0.085
Myocardial infarction	13 (9)	38 (15)	0.083
Stroke	5 (3)	12 (5)	0.658
Death	2 (1)	3 (1)	0.902

Abbreviation: PAD (peripheral artery disease).

**Table 4:** Adjusted hazard ratios for associations between immunomodulatory proteins and 2-year major adverse cardiovascular events in patients with peripheral artery disease.

	Hazard Ratio *	95% CI Lower	95% CI Upper	*p*-Value
Galectin-1	1.45	1.09	1.92	0.019
Alpha-1-Microglobulin	1.31	1.06	1.63	0.013
Galectin-9	1.35	1.02	1.78	0.028

* Adjusted for age, sex, hypertension, dyslipidemia, diabetes, past/current smoking, congestive heart failure, coronary artery disease, and previous stroke. Hazard ratios were calculated for every 0.1 pg/mL increase in plasma protein concentration. Abbreviation: CI (confidence interval).

## Data Availability

The original contributions presented in the study are included in the article; further inquiries can be directed to the corresponding author.
